# Prevalence and correlates of non-daily and daily cannabis use among persons 15 years and older in South Africa: results of a national survey in 2017

**DOI:** 10.1186/s13011-021-00364-z

**Published:** 2021-03-20

**Authors:** Shandir Ramlagan, Karl Peltzer, Supa Pengpid

**Affiliations:** 1 Department of Human and Social Capabilities, Human Sciences Research Council, Private Bag X41, Pretoria, 0001 South Africa; 2 Department of Research Administration and Development, University of Limpopo, Sovenga, 0727 South Africa; 3 ASEAN Institute for Health Development, Mahidol University, Salaya, Phutthamonthon, Nakhon Pathom, 73170 Thailand

**Keywords:** Cannabis use, Alcohol use, Drug use, Adolescents, Adults, Health variables, South Africa

## Abstract

**Background:**

The study aimed to assess the prevalence and correlates of non-daily and daily cannabis use among persons 15 years and older in South Africa.

**Method:**

In a national cross-sectional 2017 survey, 39,207 persons 15 years and older (Median = 34 years) responded to a questionnaire on cannabis use and health variables. Multinominal logistic regression was used to assess the determinants of nondaily and daily cannabis use among the general population and logistic regression for the determinants of daily cannabis use among active cannabis users.

**Results:**

Results indicate that 5.0% of the participants engaged in non-daily and 2.8% in daily cannabis use in the past 3 months. In adjusted multinomial logistic regression analysis, male sex, Grade 8–11 education, Coloureds, alcohol use disorder, never married, and other drug use were positively associated with daily cannabis use while not in not labour force was negatively associated with daily cannabis use. Moreover, male sex, never married, alcohol use disorder, and other drug use were positively, while physical multimorbidity was negatively associated with nondaily cannabis use. In adjusted logistic regression, compared to nondaily cannabis users, daily cannabis users were more likely male and were less likely not in the labour force and were less likely using other drugs.

**Conclusion:**

About one in ten participants had used cannabis in the past 3 months in South Africa. Several sociodemographic and health indicators were identified that were associated with non-daily and/or daily cannabis use.

## Introduction

*Cannabis* is the genus name of a plant from the Cannabaceae family [1] and cannabis is also the name associated with the drug produced from this plant [2]. Common names of cannabis in South Africa also include dagga, zol [3] and marijuana [4]. Globally, cannabis is the most commonly used drug [5]. The World Drug Report states that from 2010 to 2016, the increase in cannabis use appears to have been greatest in Africa and Asia [5]. According to statistics from the United Nations Office on Drugs and Crime (UNODC), globally in 2018, the annual prevalence of cannabis use was 3.86% [6].

In terms of cannabis usage in different countries around the world, the prevalence of past 12-month cannabis use in Australia was 6% in 2012 [7] and the annual prevalence in 2016 was 10.4% [2]. Annual prevalence of cannabis use in the United States of America was 18.4% in 2017, 14.03% in New Zealand in 2017, 9.3% in Uruguay in 2014, 2.5% in Brazil in 2016 and 3.3% in Bangladesh in 2004 [6] to state a few. In terms of UNODC cannabis statistics for South Africa, the organisation reports an annual cannabis prevalence at 3.65% in 2011, and that 43.3% of people in treatment facilities in 2018 were admitted for cannabis use [6].

In terms of a national population-based South African study, a 2012 study on persons 15 years and older reported the prevalence of past 3-month cannabis use at 4.0% [8]. More recent South African data on cannabis use can be found from treatment centre data that shows that cannabis is the most used drug in South African treatment facility cases in the second half of 2016 [9] and the first half of 2017 [10]. The treatment facility reports, although they are limited as they only record the number of patients seeking treatment and the substances they are seeking treatment for. There is a lack of more recent national population-based data on the prevalence and correlates of nondaily and daily cannabis use in South Africa.

Risk factors for cannabis use or cannabis use disorder include sociodemographic factors and health variables. Sociodemographic risk factors for cannabis use include male sex [7, 11–14], younger age [7, 11, 13], divorced, separated, never married [15], unemployed, living without a partner, higher education, and lower education [11]. Health variable risk factors include other illicit drug use [11, 12], alcohol use disorder [7, 11, 12, 14], psychological distress [16, 17], having no chronic conditions [12] and less frequent primary health care utilization [18, 19].

Cannabis is not indigenous to southern Africa; it is the most common illicit substance used in South Africa; it is inexpensive; it is easy to produce with South Africa being a large producer of cannabis, and the law prohibiting possession is infrequently enforced [3]. In terms of law, South Africa followed international treaties making cannabis usage a criminal offense [4], with the Prevention and Treatment of Substance Abuse Act 70 of 2008 speaking to the National Drug Master Plan on supply, demand and harm reduction [20]. These laws focused more towards drug trafficking [4] and thus not towards the individual user.

Epidemiological population-based surveys are needed to target interventions to prevent harmful cannabis use. The study aimed to assess the prevalence and correlates of non-daily and daily cannabis use among persons 15 years and older in South Africa.

## Methods

### Study design and participants

A cross-sectional, nationally representative South African National HIV Prevalence, Incidence, Behaviour and Communication Survey of persons 15 years and older in 2017 living in South Africa was analysed. This multistage stratified random cluster population-based household sample is described elsewhere [21]. In brief, the 2015 national population sampling frame [22] was utilized to draw 1000 small area layers (SALs) that were stratified by South Africas nine provinces, and locality types. In each of the 1000 SALs, 15 households were randomly selected to participate and all individuals living in the selected household that slept there the night before were invited to participate. It is important to point out that this paper utilized racial categorization where “Coloured” or mixed race is defined as children born to parents of Black African and either White and/or Indian/Asian race groups as per South Africa’s Apartheid government’s Act 30 of 1950. This is done to correct the inequalities of the previous apartheid regime.

### Study procedures

Participants were handed an informed consent form to read together with a trained interviewer. If the participant agreed to participate, they signed the consent form. For those younger than 18 years old, parental consent was sought together with youth assent. If either the youth or the parent did not sign the assent/consent form, no interview was conducted. All interviews were done in private and kept confidential. Data were collected electronically on a tablet using CSPro software. Data collection started in December 2016 and ended in February 2018. The household response rate was 82.2% and the individual response rate to be interviewed was 93.6% [21]. The participants did not receive any payment or gifts for the interview. For this paper, we restricted the sample to those with complete cannabis use measurement, 5.1% had missing cannabis values.

### Measures

*Non-daily and daily cannabis use* was assessed using the question: “In the past three months, how often have you used cannabis (dagga, marijuana, pot, grass, hash, etc.)?” from the “Alcohol, Smoking and Substance Involvement Screening Test (ASSIST)” [23]. Response options were “Never, once or twice, monthly, weekly, or almost daily.” “Non-daily” was defined as “once or twice, monthly, or weekly” and “almost daily” as “daily” cannabis use.

*Past three months, other drug use* was assessed with six items (cocaine, amphetamine, inhalants, sedatives, hallucinogens, and opiates) from the (ASSIST)” [23]. The six items were summed to define any other drug use in the past 3 months. Cronbach’s alpha for the 6-item other drug use measure was 0.97 in this sample.

*Alcohol use disorder* was assessed using the Alcohol Use Disorders Identification Test (AUDIT) [24] and was scored as in a previous survey in South Africa [25]. Among adults (20 years and above), a cut-off score of 8 or more [24] and among adolescents (15–19 years), 5 or more [26] for classifying an alcohol use disorder. (Cronbach alpha 0.87 in this sample)*.*

Sociodemographic factors included age (15–29, 30–44, and 45 or more years), sex (male, female), highest educational level (pre-school or Gr R, grades 1–12, further studies incomplete, diploma/undergraduate degree/post school completed, further degree completed), marital status (married, never married, divorced or separated, widow or widower), population group or ethnicity (African, White, Coloured, Indian or Asian, other), employment status (unemployed, sick or disabled and unable to work, student or pupil or learner, employed of self-employed, other) and residence status (rural informal or tribal areas, rural farms, urban) [21].

Psychological distress was assessed with the Kessler Psychological Distress Scale (K10), with scores 20 or more indicating psychological distress [27]. Cronbach’s alpha for the K10 was 0.92 in this sample.

*Physical multimorbidity* was assessed with self-reported health care provider diagnosed hypertension, diabetes, HIV, cancer and heart disease (Yes, No).

*Health care utilization* was sourced from the question: When was the last time you went to see a health professional (doctor, nurse, traditional healer, etc.)? Response options were 1 = within the last 6 months, 2 = more than 6 months but not more than a year ago, 3 = more than 1 year ago, and 4 = never (coded 1 = within the last 6 months and 0 = 2–4).

### Data analysis

All statistical analyses were conducted using STATA software version 14.0 (Stata Corporation, College Station, TX, USA). The data were weighted to make the sample representative of the target population in South Africa. Descriptive statistics were used to summarize the sample and cannabis use prevalence characteristics. Unadjusted and adjusted (including variables significant at *p* < 0.05 in univariate analysis) multinomial logistic regression was used to predict nondaily and daily cannabis use, with no past 3-month cannabis use as the reference category. In addition, unadjusted and adjusted (including variables significant at *p* < 0.05 in univariate analysis) logistic regression was used among active (past 3 months) cannabis users to predict daily versus nondaily cannabis use. Taylor linearization methods were applied to account for the complex study design and the sampling weight. Results from multinomial logistic regression analyses are reported as relative risk ratios and from logistic regression as odds ratios (ORs), and 95% confidence intervals (CIs). Missing values (< 1.8% for any study variable) were excluded and *p* < 0.05 considered significant.

## Results

### Characteristics of the sample and cannabis use

The sample comprised 39,207 persons 15 years and older (Median = 34 years, interquartile range = 25–48), 48.3% were men, and 51.7% were women, 36.1% had Grade 12 or more education, 62.5% had never been married, and 79.3% were Black African by population group or ethnicity. More than one in three participants (35.6%) were employed or self-employed, 69.0% lived in urban areas, 20.5% reported psychological distress, 4.9% physical multimorbidity, and 47.3% past 6-month health care utilization. More than one in ten respondents (10.3%) had an alcohol use disorder, and 2.8% used drugs other than cannabis in the past 3 months. Five percent of the participants engaged in non-daily and 2.8% in daily cannabis use in the past 3 months (Table 1).

**Table 1:** Sample characteristics and distribution of cannabis use among persons 15 years and older in South Africa, 2017

Variable	Sample	Past 3-month cannabis use		
		Never (*N* = 36,503)	Non-daily (*N* = 1747)	Almost daily (*N* = 957)
	N (%)	%	%	%
All	39,207	92.2	5.0	2.8
Sex				
Female	23,102 (51.7)	96.5	3.1	0.4
Male	16,105 (48.3)	87.7	7.1	5.3
Age in years				
15–29	15,524 (38.1)	90.5	6.3	3.3
30–44	10,604 (32.4)	91.6	5.2	3.3
45 or more	13,079 (29.5)	95.2	3.2	1.6
Education				
Grade 0–7	16,887 (37.3)	93.6	4.6	1.8
Grade 8–11	9876 (26.6)	89.9	5.5	4.6
Grade 12 or more	12,367 (36.1)	92.6	5.0	2.5
Marital status				
Married	10,514 (28.9)	95.6	3.1	1.3
Separated/divorced/widowed	3739 (8.6)	96.0	2.7	1.3
Never married	24,946 (62.5)	90.1	6.2	3.7
Population group				
African Black	30,670 (79.3)	92.3	4.9	2.8
White	1924 (9.0)	93.7	4.8	1.4
Coloured	4304 (8.8)	89.7	6.0	4.2
Indian or Asian	2309 (2.9)	93.9	4.9	1.2
Employement status				
Employed/self-employed	11,937 (35.6)	91.9	4.7	3.4
Unemployed	20,649 (49.6)	92.1	5.1	2.8
Not in labour force	6571 (14.8)	93.4	5.4	1.2
Residence				
Rural informal (tribal areas)	13,584 (26.0)	93.4	4.8	1.8
Rural (farms)	4266 (5.0)	92.3	5.2	2.5
Urban	21,357 (69.0)	91.8	5.1	5.1
Alcohol use disorder				
No	36,067 (89.7)	93.8	4.2	2.0
Yes	3087 (10.3)	78.3	11.9	9.8
Other drugs				
No	37,991 (97.2)	94.1	3.5	2.4
Yes	1084 (2.8)	32.4	55.9	11.7
Psychological distress				
No	31,304 (79.5)	92.4	4.9	2.7
Yes	7751 (20.5)	91.4	5.4	3.2
Physical multimorbidity				
0	29,394 (77.2)	91.3	5.5	3.2
1	7056 (17.9)	94.6	3.8	1.6
2 or more	2279 (4.9)	97.7	2.0	0.4
Health care utilization				
No	20,823 (52.7)	90.8	5.7	3.6
Yes	18,232 (47.3)	93.8	4.3	1.9

### Associations with non-daily and daily cannabis use among the general population

In adjusted multinomial logistic regression analysis, male sex, Grade 8–11 education, Coloureds, alcohol use disorder, never married, and other drug use were positively associated with daily cannabis use while not in not labour force was negatively associated with daily cannabis use. Male sex, never married, alcohol use disorder, and other drug use were positively, while physical multimorbidity was negatively associated with nondaily cannabis use. In addition, in univariate multinomial logistic regression, urban residence was positively and Whites and Indians or Asians were negatively associated with daily cannabis use (Tables 2 and 3).

**Table 2:** Simple multinomial regression with cannabis use

Variable	Non-daily cannabis use		Almost daily cannabis use	
	Crude RRR (95% CI)	*P*-value	Crude RRR (95% CI)	*P*-value
Sex				
Female	1 (Reference)		1 (Reference)	
Male	2.52 (2.06, 3.09)	< 0.001	13.35 (9.11, 19.58)	< 0.001
Age in years				
45 or more	1 (Reference)		1 (Reference)	
30–44	1.69 (1.36, 2.09)	< 0.001	2.06 (1.43, 2.96)	< 0.001
15–29	2.08 (1.68, 2.57)	< 0.001	2.08 (1.49, 2.91)	< 0.001
Education				
Grade 0–7	1 (Reference)		1 (Reference)	
Grade 8–11	1.25 (1.01, 1.57)	0.042	2.60 (1.97, 3.44)	< 0.001
Grade 12 or more	1.09 (0.89, 1.35)	0.404	1.36 (1.02, 1.81)	0.037
Marital status				
Married	1 (Reference)		1 (Reference)	
Separated/divorced/widowed	0.87 (0.63, 1.22)	0.427	0.97 (0.47, 2.02)	0.944
Never married	2.12 (1.71, 2.62)	< 0.001	3.05 (2.07, 4.51)	< 0.001
Population group				
African Black	1 (Reference)		1 (Reference)	
White	0.97 (0.71, 1.32)	0.836	0.50 (0.27, 0.94)	0.032
Coloured	1.27 (0.97, 1.66)	0.086	1.54 (1.13, 2.08)	0.006
Indian or Asian	0.98 (0.95, 1.47)	0.913	0.42 (0.24, 0.73)	0.002
Employement status				
Employed/self-employed	1 (Reference)		1 (Reference)	
Unemployed	1.07 (0.88, 1.29)	0.499	0.83 (0.63, 1.09)	0.170
Not in labour force	1.11 (0.86, 1.43)	0.414	0.35 (0.21, 0.57)	< 0.001
Residence				
Rural informal (tribal areas)	1 (Reference)		1 (Reference)	
Rural (farms)	1.12 (0.83, 1.51)	0.472	1.39 (0.85, 2.29	0.187
Urban	1.09 (0.86, 1.37)	0.474	1.77 (1.22, 2.59)	0.003
Alcohol use disorder				
No	1 (Reference)		1 (Reference)	
Yes	3.88 (2.68, 4.28)	< 0.001	5.88 (4.54, 7.63)	< 0.001
Other drugs				
No	1 (Reference)		1 (Reference)	
Yes	46.89 (34.72, 63.33)	< 0.001	13.96 (9.04, 21.54)	< 0.001
Psychological distress				
No	1 (Reference)		1 (Reference)	
Yes	1.11 (0.86, 1.43)	0.412	1.22 (0.87, 1.72)	0.254
Physical multimorbidity				
0	1 (Reference)		1 (Reference)	
1	0.66 (0.53, 0.82)	< 0.001	0.50 (0.33, 0.76)	< 0.001
2 or more	0.34 (0.21, 0.53)	< 0.001	0.11 (0.04, 0.33)	< 0.001
Health care utilization				
No	1 (Reference)		1 (Reference)	
Yes	0.72 (0.61, 0.85)	< 0.001	0.52 (0.39, 0.69)	< 0.001

RRR Relative Risk Ratio, *CI* Confidence Interval

**Table 3:** Multiple multinomial regression with non-daily and daily cannabis use (reference no past 3-month cannabis use)

Variable	Non-daily cannabis use		Almost daily cannabis use	
	Adjusted RRR (95% CI)	*P*-value	Adjusted RRR (95% CI)	*P*-value
Sex				
Female	1 (Reference)		1 (Reference)	
Male	2.20 (1.77, 2.75)	< 0.001	10.55 (6.88, 16.18)	< 0.001
Age in years				
45 or more	1 (Reference)		1 (Reference)	
30–44	1.06 (0.81, 1.38)	0.665	0.92 (0.61, 1.38)	0.685
15–29	1.09 (0.80, 1.49)	0.587	0.92 (0.60, 1.39)	0.677
Education				
Grade 0–7	1 (Reference)		1 (Reference)	
Grade 8–11	1.14 (0.88, 1.49)	0.322	1.75 (1.26, 2.44)	< 0.001
Grade 12 or more	1.02 (0.76, 1.36)	0.899	0.99 (0.70, 1.40)	0.974
Marital status				
Married	1 (Reference)		1 (Reference)	
Separated/divorced/widowed	0.95 (0.62, 1.45)	0.800	1.08 (0.51, 2.28)	0.843
Never married	2.05 (1.54, 2.71)	< 0.001	3.00 (1.97, 4.59)	< 0.001
Population group				
African Black	1 (Reference)		1 (Reference)	
White	1.58 (1.06, 2.24)	0.024	0.88 (0.42, 1.84)	0.730
Coloured	1.25 (0.90, 1.72)	0.189	1.52 (1.09, 2.11)	0.013
Indian or Asian	1.27 (0.76, 2.12)	0.368	0.59 (0.31, 1.13)	0.112
Employement status				
Employed/self-employed	1 (Reference)		1 (Reference)	
Unemployed	1.08 (0.83, 1.39)	0.570	0.89 (0.64, 1.23)	0.470
Not in labour force	0.97 (0.68, 1.39)	0.879	0.32 (0.17, 0.61)	< 0.001
Residence				
Rural informal (tribal areas)	1 (Reference)		1 (Reference)	
Rural (farms)	1.00 (0.70, 1.44)	0.990	0.94 (0.54, 1.64)	0.818
Urban	1.00 (0.77, 1.30)	0.983	1.45 (0.92, 2.28)	0.106
Alcohol use disorder				
No	1 (Reference)		1 (Reference)	
Yes	2.89 (2.12, 3.78)	< 0.001	3.07 (2.30, 4.09)	< 0.001
Other drugs				
No	1 (Reference)		1 (Reference)	
Yes	52.29 (36.17, 75.68)	< 0.001	16.44 (9.75, 27.72)	< 0.001
Physical multimorbidity				
0	1 (Reference)		1 (Reference)	
1	0.86 (0.64, 1.15)	0.311	0.87 (0.53, 1.41)	0.562
2 or more	0.47 (0.29, 0.78)	0.003	0.24 (0.07, 0.79)	0.020
Health care utilization				
No	1 (Reference)		1 (Reference)	
Yes	0.94 (0.78, 1.13)	0.518	0.77 (0.56, 1.05)	0.099

*RRR* Relative Risk Ratio, *CI* Confidence Interval

### Associations with daily cannabis use among active cannabis users

In adjusted logistic regression, compared to nondaily cannabis users, daily cannabis users were more likely male and were less likely not in the labour force and were less likely using other drugs. In addition, in univariate analysis, grades 8–11, urban residence, and alcohol use disorder were positively, and Whites and Indians or Asians and health care utilization were negatively associated with daily cannabis use (Table 4).

**Table 4:** Logistic regression with daily cannabis use among active cannabis users (*N* = 1728)

Variable	Crude OR (95% CI)	*P*-value	Adjusted OR (95% CI)	*P*-value
Sex				
Female (23.4%)	1 (Reference)		1 (Reference)	
Male (76.6%)	5.29 (3.40, 8.25)	< 0.001	3.88 (2.40, 6.28)	< 0.001
Age in years				
45 or more (18.3%)	1 (Reference)		–	
30–44 (35.1%)	1.22 (0.80, 1.86)	0.351		
15–29 (46.7%)	1.00 (0.68, 1.47)	0.999		
Education				
Grade 0–7 (30.9%)	1 (Reference)		1 (Reference)	
Grade 8–11 (34.6%)	2.07 (1.46, 2.94)	< 0.001	1.25 (0.82, 1.99)	0.298
Grade 12 or more (34.5%)	1.24 (0.86, 1.79)	0.248	0.90 (0.57, 1.44)	0.670
Marital status				
Married (16.3%)	1 (Reference)		–	
Separated/divorced/widowed (4.4%)	1.12 (0.50, 2.47)	0.788		
Never married (79.3%)	1.44 (0.92, 2.25)	0.107		
Population group				
African Black (78.9%)	1 (Reference)		1 (Reference)	
White (7.2%)	0.52 (0.27, 1.00)	0.049	0.65 (0.32, 1.32)	0.236
Coloured (11.6%)	1.21 (0.83, 1.76)	0.310	1.12 (0.71, 1.78)	0.618
Indian or Asian (2.3%)	0.43 (0.24, 0.75)	0.003	0.54 (0.30, 0.99)	0.046
Employement status				
Employed/self-employed (37.2%)	1 (Reference)		1 (Reference)	
Unemployed (50.3%)	0.77 (0.55, 1.09)	0.137	0.85 (0.57, 1.26)	0.421
Not in the labour force (12.5%)	0.31 (0.18, 0.55)	< 0.001	0.33 (0.16, 0.66)	0.002
Residence				
Rural informal (tribal areas) (22.0%)	1 (Reference)		1 (Reference)	
Rural (farms) (5.0%)	1.25 (0.77, 2.04)	0.372	0.96 (0.55, 1.69)	0.892
Urban (73.1%)	1.63 (1.12, 2.38)	0.012	1.54 (0.98, 2.45)	0.068
Alcohol use disorder				
No (71.3%)	1 (Reference)		1 (Reference)	
Yes (28.7%)	1.74 (1.24, 2.43)	< 0.001	1.26 (0.85, 1.85)	0.240
Other drugs				
No (74.9%)	1 (Reference)		1 (Reference)	
Yes (25.1%)	0.30 (0.19, 0.47)	< 0.001	0.42 (0.26, 0.66)	< 0.001
Psychological distress				
No (77.4%)	1 (Reference)		–	
Yes (22.6%)	1.10 (0.73, 1.65)	0.646		
Physical multimorbidity				
0 (86.1%)	1 (Reference)		–	
1 (12.4%)	0.76 (0.47, 1.24)	0.276		
2 or more (1.5%)	0.32 (0.10, 1.06)	0.062		
Health care utilization				
No (62.5%)	1 (Reference)		1 (Reference)	
Yes (37.5%)	0.72 (0.51, 1.00)	0.048	0.82 (0.58, 1.17)	0.276

*OR* Odds Ratio, *CI* Confidence Interval

## Discussion

This paper utilized data from a large national population-based household survey in 2017 to assess the prevalence and correlates of non-daily and daily cannabis use among persons 15 years and older living in South Africa. The study found that 5 % of the participants engaged in non-daily and 2.8% in daily cannabis use in the past 3 months. This finding shows a considerable increase from previous studies where the prevalence of past 3 months cannabis use was found to be 4% [8] and 3.3% [4] during the 2012 and 2008 surveys which used the same survey methodology. During the same period as this survey, treatment study data showed that cannabis is the most used drug in South African treatment facility cases [9, 10].

The 2017 survey findings almost doubled that of the survey done in 2012 and could be attributed to the ease of access to cannabis and it being cost effective [3, 8]. It is also important to mention that previous studies have stated that laws on cannabis restrictions were in place during the survey period, but the enforcement of those laws were not geared to the single end user but rather to the drug trafficker [3, 4, 20]. Although cannabis use was illegal during the survey period, the medical benefits of cannabis [2] have been voiced and decriminalization propagated in South Africa [20]. All of this coupled together could potentially seem as although cannabis is more tolerated in South African society, thus leading to increased usage.

Evidence from this analysis shows that when compared to females, males were about 11 times more likely to be daily cannibis users. The finding that males are significantly associated with cannabis use concurs with previous published studies and South African treatment facility reports [3, 4, 7, 8, 11–14, 28, 29]. Although the data from our study is household data from the general population and does not ask for motivation for cannabis use, the treatment facility literature suggests that if more males are seeking treatment for their cannabis use than interventions are needed more especially for males.

Although those participants having Grade 8–11 education made up a quarter of the study population aged 15 years and older, this study found that they are positively associated with daily cannabis use. This finding is in contravention of the 2008 finding which showed that having a grade 8–11 educational level was protective for men [4] nor the 2012 finding which found no association between cannabis use and educational level [8]. This change from previous years is of concern as studies have shown that increasing cannabis use was associated with an increasing risk of leaving school without qualifications [30, 31]. These increases could be attributed to the ease with which cannabis can be obtained and law enforcements focus on drug traffickers rather than the end user [4]. Interestingly, persons not in the labour force had lower odds of daily cannabis use, which is a very promising outcome.

Respondents from the Coloured population group had significantly higher odds, of one and a half times, for daily cannabis use. This finding parallels previous cannabis and other drug studies [3, 4, 8, 32]. The coloured population could be experiencing increased stressors [33] which could account for the positive association. These stressors emanate from Apartheid era policies which marginalised non-white population groups in South Africa by limiting their access to better education, income generation, better living conditions, and so on. This generational impoverishment could have led to increased cannabis use and thus targeted interventions using an Intervention Mapping Framework [34] to find the most appropriate interventions are required to remedy the injustices of the past.

As found in previous research [7, 15], compared to individuals who never married, those who were married was found protective against cannabis use. It is possible that people who never married engage in daily cannabis use because of being disadvantaged in forming relationships [7] as this study found that they were two times more likely to be non-daily cannabis users and three times more likely to be daily cannabis users when compared to those who are married. The study showed that alcohol use disorder and other drug use were highly significantly positively associated with past 3-month daily and nondaily cannabis use, where those that used other drugs were 52 times more likely to be non-daily cannabis users and 16 times more likely to be daily cannabis users. These finds are similar to other research where it would seem that cannabis and alcohol usage are interlinked [7, 11, 12, 14, 35] as well as cannabis and other drug usage [11, 12]. Interestingly, the adjusted logistic regression showed that among cannabis users, other drug use had lower odds of daily cannabis use and could be due to other drug users not considering cannabis as their primary drug. These findings do suggest that interventions regarding reducing cannabis abuse, alcohol abuse, and other drug usage should be integrated.

Those respondents who had multiple diseases or conditions were negatively associated with non-daily cannabis use. Similar results were found among middle-aged and older adults in the United States [12]. This result seems to indicate that cannabis use in this study was more likely used for recreational than medicinal purposes for multiple chronic conditions. Unlike some previous research [16, 17], this survey did not show an association between psychological distress and non-daily or daily cannabis use. In line with previous studies on risky alcohol use [18, 19], this study found an association between less frequent primary health care utilization and non-daily and daily cannabis use. It is possible that those with cannabis use more frequently attend hospital or emergency medical services.

### Study limitations

The study was limited by its cross-sectional design and self-report of data, including cannabis use. The self-report of cannabis use may led to biased responses. Cannabis and other drug use were only assessed with a shortened version of the ASSIST, not allowing us to report on cannabis and other drug use disorders, as well as tobacco use disorders. However, it has been estimated [36] that one-third of almost daily cannabis users would fulfil the criteria of DSM III cannabis dependence. Another study limitation was that there was no control over the Type I error rate with multiple tests. Thus, the chance for a Type I error is larger than 0.05. Futhermore, the motivation of cannabis use, such as medicinal or recreactional, was not assessed, and should be included in future studies.

## Conclusions

In this large national population-based survey among persons 15 and older in 2017 in South Africa, almost one in ten participants engaged past 3-month cannabis use. Several sociodemographic factors (male sex, never married, having Grade 8–11 education, and Coloureds) and health indicators (alcohol use disorder, other drug use and no physical multimorbidity) were identified that were associated with non-daily and/or daily cannabis use. Among active cannabis users, other drug use had lower odds of daily cannabis use. It is recommended that reasons or motivations for cannabis use be investigated to fully understand its usage and then make informed policy recommendations. Given that school going children, Grades 8–11 have now started using cannabis compared to previous years, literature, requires immediate intervention to curb the usage and thus ensure these youth stay in school. Among the Coloured population group, targeted inteventions are required to remedy the injustices of South Africa’s Apartheid past. Substance abuse policies need to be integrated to take all substances, including cannabis, alcohol, and illicit drugs, into consideration as this study as well as the literature show that they are interlinked and thus require an overarching integrated intervention with greater emphasis on illicit drug use.

## Data Availability

The dataset used and/or analysed during the current study is available from the. Human Sciences Research Council [distributor] 2020. 10.14749/1585345902.

## Abbreviations

ASSIST: Alcohol, Smoking and Substance Involvement Screening Test
AUDIT: Alcohol Use Disorders Identification Test;
DSM: Diagnostic and Statistical Manual of Mental Disorders
K10: Kessler Psychological Distress Scale
SALs: Small area layers
